# Predicting User Engagement in Health Misinformation Correction on Social Media Platforms in Taiwan: Content Analysis and Text Mining Study

**DOI:** 10.2196/65631

**Published:** 2025-01-23

**Authors:** Hsin-Yu Kuo, Su-Yen Chen

**Affiliations:** 1 Department of Educational Psychology and Counseling National Tsing Hua University Hsinchu Taiwan; 2 Institute of Learning Sciences and Technologies National Tsing Hua University Hsinchu Taiwan

**Keywords:** health misinformation, misinformation correction, fact-checking, content analysis, text mining, fuzzy-trace theory, social media, large language models, user engagement, health communication

## Abstract

**Background:**

Health misinformation undermines responses to health crises, with social media amplifying the issue. Although organizations work to correct misinformation, challenges persist due to reasons such as the difficulty of effectively sharing corrections and information being overwhelming. At the same time, social media offers valuable interactive data, enabling researchers to analyze user engagement with health misinformation corrections and refine content design strategies.

**Objective:**

This study aimed to identify the attributes of correction posts and user engagement and investigate (1) the trend of user engagement with health misinformation correction during 3 years of the COVID-19 pandemic; (2) the relationship between post attributes and user engagement in sharing and reactions; and (3) the content generated by user comments serving as additional information attached to the post, affecting user engagement in sharing and reactions.

**Methods:**

Data were collected from the Facebook pages of a fact-checking organization and a health agency from January 2020 to December 2022. A total of 1424 posts and 67,378 corresponding comments were analyzed. The posts were manually annotated by developing a research framework based on the fuzzy-trace theory, categorizing information into “gist” and “verbatim” representations. Three types of gist representations were examined: risk (risks associated with misinformation), awareness (awareness of misinformation), and value (value in health promotion). Furthermore, 3 types of verbatim representations were identified: numeric (numeric and statistical bases for correction), authority (authority from experts, scholars, or institutions), and facts (facts with varying levels of detail). The basic metrics of user engagement included shares, reactions, and comments as the primary dependent variables. Moreover, this study examined user comments and classified engagement as cognitive (knowledge-based, critical, and bias-based) or emotional (positive, negative, and neutral). Statistical analyses were performed to explore the impact of post attributes on user engagement.

**Results:**

On the basis of the results of the regression analysis, risk (β=.07; *P*=.001), awareness (β=.09; *P*<.001), and facts (β=.14; *P*<.001) predicted higher shares; awareness (β=.07; *P*=.001) and facts (β=.24; *P*<.001) increased reactions; and awareness (β=.06; *P*=.005), numeric representations (β=.06; *P*=.02), and facts (β=.19; *P*<.001) increased comments. All 3 gist representations significantly predicted shares (risk: β=.08; *P*<.001, awareness: β=.08; *P*<.001, and value: β=.06; *P*<.001) and reactions (risk: β=.04; *P*=.007, awareness: β=.06; *P*<.001, and value: β=.05; *P*<.001) when considering comment content. In addition, comments with bias-based engagement (β=–.11; *P*=.001) negatively predicted shares. Generally, posts providing gist attributes, especially awareness of misinformation, were beneficial for user engagement in misinformation correction.

**Conclusions:**

This study enriches the theoretical understanding of the relationship between post attributes and user engagement within web-based communication efforts to correct health misinformation. These findings provide a foundation for designing more effective content approaches to combat misinformation and strengthen public health communication.

## Introduction

### Background

Health misinformation negatively impacts individuals’ and society’s adaptive response to a health crisis [1,2]. As a communication tool, social media has worsened the problem by speeding up the spread of misinformation and strengthening people’s existing beliefs [3-5]. Conversely, social media platforms can be effectively used for health communication to disseminate accurate information by correcting misinformation and engaging users by providing verified health advice [6-9]. Many organizations devote efforts to correcting misinformation and releasing them on social media. However, the efficacy of correction is sometimes limited for various reasons, such as people feeling uncomfortable with the knowledge gap when retracting misinformation [10]. Moreover, in an increasingly complex health information environment, people are flooded with knowledge they may not be able to use correctly [11]. In this context, the conditions and message designs that make misinformation correction an effective countermeasure have been examined in experimental studies [6,12,13]. As an interactive digital environment, social media also allows researchers to access data on user responses to health communication, obtaining results from different perspectives [7-9,14].

In this study, we investigate how to enhance the effectiveness of misinformation correction based on real-world data. We selected 2 representative Facebook fan pages in Taiwan (Facebook, Meta Platforms, Inc) that provide rich health misinformation corrections in post forms. Users engage with the content provided by these 2 organizations and other users’ comments. In general, correction is a remedy that offers accurate information to counteract misinformation presented in media [10]. According to the observed data, the definition of health misinformation correction in this study is the content with the intent to resist health-related misinformation. This includes clarifications of health misinformation, fact-checking that covers the verification process, and content that addresses potential misinformation while not directly correcting a specific claim (examples are provided in Table S1 in Multimedia Appendix 1). The topics covered include diseases; health care; food safety; and issues extending from the COVID-19 pandemic, such as public welfare and information security. Then, we need a theoretical framework to identify the posts’ attributes (called features or characters in some studies [7,9]). The fuzzy-trace theory (FTT) provides a framework that differentiates between 2 cognitive representations of a message, “gist” (top-down meaning) and “verbatim” (detailed information) [15], helping us focus on analyzing textual meaning in posts rather than merely considering surface attributes such as length and tone. The theory was also used to explore physician-patient, health, and scientific communication [7,16,17].

The responses social media users have when they see posts, particularly those explicitly displayed on social media, are referred to as user engagement in this study. These are important metrics for examining the impacts of post attributes in many studies [8,9,14]. This study explored user engagement in 2 dimensions. The first is based on the metrics Facebook provides (sharing, reacting, and commenting), which are the main focus of the predictions in this research. The second is to examine the content users create when they comment. We assume that users engage with different intentions in their comments, and this is reflected in the comment content, which is presented as additional information accessible to other users. Then, this study proposes a framework to classify the engagement categories in comments and examine if this content affects the other 2 metrics: sharing and reacting.

We then introduced prior work about health communication on social media, explained the research gap, and explored the perspective of FTT to identify post attributes and the relationship between post attributes and user engagement in this study.

### Prior Work

In previous studies, the acceptance of misinformation correction across different contexts and content types has been examined through experiments. The variables often examined include simple versus detailed or factual elaboration [12,13,18]; correction sources [6,12,13,19]; and information content such as quality, intensity, or emphasis [18,20,21].

Another approach in the context of social media involves collecting data from these platforms, labeling them manually or automatically, and analyzing them. A study using FTT as a framework explored news reports about measles and vaccines and expressed the opinions of vaccination proponents or opponents as the gist. In this study, data were coded to predict the likelihood of news sharing on Facebook. The regression model included other variables, such as statistics and stories, but only the gist could significantly predict news sharing [14]. Health communication efforts increased significantly during the COVID-19 pandemic, allowing for analysis through publicly available data. A study of 1816 posts from hospitals in Taiwan during the first 4 months of the COVID-19 pandemic found that specific social media strategies significantly increased user engagement, indicating the potential to improve health literacy among health care organizations [7]. Such studies have also observed that users inevitably share misinformation through comments when discussing health issues on social media. A study analyzed 1534 comments from 2 Facebook groups discussing mental health symptoms between January 2019 and December 2021. It found that 26.1% of the comments contained medically inaccurate information, specifically regarding complementary and alternative medicine (60.3%) [22]. By observing long-term trends, it is also possible to track changes in the quality of health communication and user attitudes. An analysis of 12,553 COVID-19 vaccine fact-checking posts and 122,362 comments from January 2020 to March 2022 showed increased analytical thinking and confidence in the posts, alongside decreased tentativeness among comments, showing an evolution in public sentiment over time [8]. Finally, a study analyzed 914 fact-checking news articles to understand how source transparency, contextual information, and vividness influence user engagement. The study found that these factors significantly affected user responses, especially likes and angry reactions, emphasizing the importance of content style and depth in capturing public attention [9]. These studies underline the critical role of social media in shaping public health narratives and emphasize the necessity of designing engaging yet accurate content during health crises.

### Research Gap

Regarding the research gap, there is a lack of health communication studies that incorporate both data and theory, and finding a theory that fits the data is not easy. To explore the effect of misinformation correction, the primary theories considered include the inoculation theory [23], elaboration likelihood model [24], heuristic-systematic model [25], and FTT [15], all of which offer different interpretations from a cognitive perspective on how misinformation correction can be effectively achieved. The FTT emphasizes the importance of conveying the essence of a message, focusing on its core meaning rather than merely summarizing or simplifying it. It highlights the need to avoid overwhelming people with excessive quantitative details. [26]. Moreover, FTT has robust cognitive neuroscience foundations, making it suitable for scientific communication and compliant with nonpersuasive fact-checking methods that prioritize reference-based corrections. Therefore, this study adopted FTT for analysis. The method of conveying essential meaning is called gist communication [17].

In addition, we proposed a framework for classifying user-generated comment content, as previous studies have rarely examined this aspect. We have included them as independent variables in the prediction model to explore whether the FTT’s gist communication remains effective even when different comments are present as additional post information.

### The FTT and Health Misinformation Correction

#### Overview

The FTT explains how we process and remember information through 2 types of cognitive representation: “gist” and “verbatim.” Gist captures the essential, top-down meaning, including causal relations and key concepts [27,28]. Verbatim captures details such as exact words and elements [15,16]. This theory was originally developed to study how individuals shape their memory and engage in reasoning and decision-making [15]. The gist is more than a mental shortcut; it is a distilled way of presenting objective facts to help individuals make health and medical decisions and navigate life [29].

According to the FTT, information containing the gist is likely to evoke motivationally relevant values that may be shared online, whether true or not [30]. This explains why people easily share misinformation that offers a clear core message and causal explanation [27]. This mechanism can be applied in fact-checking or correction as an intervention. FTT explains that providing a brief explanation has more lasting benefits than merely offering a false label [31], but offering decontextualized detailed information may be counterproductive [18]. The critical factor may be the inclusion of the gist. The gist of information triggers emotions and evokes core values [17]. Furthermore, when the content provides gist cues that connect with people’s inner motivations and values, it is more likely to catch attention and be shared [30].

Health interventions should emphasize conveying the gist of information with an organized approach to highlight essential points [32]. The next question is to identify the gist of the health misinformation correction. Different gist representations exist in diverse contexts. In a study on physician-patient communication, the gist of the effectiveness and risks of medications was extracted from communication records to examine its relationship with patient satisfaction [16]. On the basis of the FTT and important concepts discussed in misinformation research, we proposed 3 gist representations: risks associated with misinformation, awareness of misinformation, and value in health promotion. In addition, we proposed 3 verbatim representations: numeric and statistical information as a reference for correction; authority from named experts, scholars, or official institutions used as references; and facts presented with varying levels of detail. These 6 representations, abbreviated as risk, awareness, value, numeric, authority, and facts, serve as attributes of misinformation correction posts.

#### Risk: Risks Associated With Misinformation

During health crises, it is important to communicate risks accurately and effectively [32,33], as it significantly correlates with adopting preventive health behaviors [34]. The risks associated with misinformation in this study included harm to personal and public health, social panic, and legal risks of spreading misinformation.

#### Awareness: Awareness of Misinformation

Public awareness plays a crucial role in improving resilience from misinformation, including awareness of exposure to misinformation and the ability to identify it [35]. Moreover, providing awareness and warnings beforehand usually has a beneficial impact [23]. In this study, awareness of misinformation involved practical information to remind and help individuals recognize and verify misinformation.

#### Value: Value in Health Promotion

Promoting health and well-being is a primary goal of health communication [36]. When correcting misinformation, it is recommended not only to mention what is false but also to provide alternative accurate information and encourage self-affirmation [10]. Conveying public trust and confidence is vital to combat anxiety-induced acceptance of misinformation [37]. Therefore, this study identified the third gist of health promotion as the core value toward health crises, including health literacy and a positive attitude.

#### Numeric: Numeric and Statistical Information as a Reference for Correction

Scientific communication often includes numbers and statistics as objective data. People’s numeracy levels can affect their interpretations of risks, probabilities, and numerical results, leading to potential misunderstandings [38]. This study examined the use of numerical information to correct misinformation. Indeed, prior content analysis studies have considered statistical data as an attribute or characteristic of posts [9,14].

#### Authority: Authority From Named Experts, Scholars, or Official Institutions Used as References

Experts, scholars, and professional organizations are trusted sources of knowledge for authoritative communication, and health care professionals can correct health misinformation on social media [39,40]. Fact-checking organizations consult experts to improve the credibility of misinformation corrections. Thus, this study also investigated the effects of referencing authority.

#### Facts: Facts Presented With Varying Levels of Detail

People’s attention span is generally limited on social media, and the effects of detailed factual information should be evaluated. The outcomes of detailed factual elaboration and simple rebuttal have been discussed in prior research [12,41]. In this study, fact representations refer to detailed and scientific elaborations with evidence for correcting misinformation. These can be categorized into three levels: (1) those that do not target specific misinformation; (2) simple corrections involving straightforward explanations; and (3) detailed corrections encompassing comprehensive information, typically with a complete structure, including background, reasons for errors, and alternative explanations.

### User Engagement on Social Media

Social media offers benefits for health communication, such as improving the accessibility of health information and allowing users to generate and exchange information, but the quality and reliability of the shared information must be monitored [42]. The FTT suggests that information with gist cues supports reasoning and decision-making, and they are more likely to share when the cues connect their motivations or values. Facebook shares are commonly used as a metric, indicating users’ affirmation and commitment to a post [43]. In addition to sharing, users can also engage through reactions (including likes and 6 emoticons) and comments on social media platforms. A study used the common-sense model of illness self-regulation to describe humans’ cognitive and emotional systems to increase user engagement toward some attributes of illness representations on social media [44]; that is, shares, reactions, and comments were metrics driven by cognitive and emotional factors.

This study defined the basic metrics of user engagement as follows:

- Shares—user engagement in sharing, measured by share count
- Reactions—user engagement in reacting, measured by summing up clicks of likes and 6 other emoticons
- Comments—user engagement in commenting, measured by comment count

Metrics such as shares, reactions, and comments were commonly used in health communication studies to identify factors that increase user engagement [44-46]. However, user engagement is not always positive, especially in comments, which often contain errors, biases, and uncivil behavior [22,47,48]. The phenomenon of echo chambers on social media refers to selective exposure and confirmation bias, which limit diverse perspectives and foster polarized groups [3]. A study distinguishes between 2 belief systems among users: scientific and conspiracy [49]. Some studies have suggested that user-generated comments influence other users’ perceptions of posts. For example, it was found that the perceived quality of journalistic content being commented on decreased due to a lack of reasoning and incivility in user comments [47]. When exposed to critical comments about a fake news article, people are less likely to share or comment positively on the article [50]. A study reported that positive comments influenced people’s perceptions of a company’s reputation in crisis [51]. However, well-informed individuals often remain quiet on certain issues, whereas misinformed individuals tend to be more vocal online [52].

This study categorized comments into cognitive and emotional engagement [44]. For cognitive engagement, we were concerned with the conspiracy belief group and its counterpart, the scientific group [49]. We observed the data potentially driven by conspiracy belief as bias-based engagement and identified 2 types of engagement driven by scientific belief: one focused on knowledge itself, called knowledge-based engagement, and the other on misinformation issues, called critical engagement. Furthermore, we observed user engagement driven by emotions, which can be divided into positive, negative, and neutral emotional engagement according to the positive-negative valence framework [53]. We classified comments into one of six categories, which are further defined as follows:

1. Knowledge-based engagement. This study observed users’ knowledge-related behaviors on social media driven by scientific beliefs, which include providing additional information, clarifying facts, and asking questions.
2. Critical engagement. Developing critical thinking skills and pragmatic skepticism is crucial for citizens to combat misinformation [35]. Unlike knowledge-based engagement, which focuses on knowledge itself, critical engagement identified in this study focused on discussing misinformation by offering warnings or explanations.
3. Bias-based engagement. Bias-based engagement refers to users disseminating biased content driven by conspiracy beliefs, including misinformation, incorrect inferences, logical fallacies, or conspiracy theories, potentially undermining information reliability and impeding evidence-based discourse.
4. Positive emotional engagement. Positive emotional engagement is characterized by users expressing positive emotions, such as joy, happiness, and excitement.
5. Negative emotional engagement. Negative emotional engagement is characterized by users expressing negative emotions, such as anger, fear, sadness, or frustration.
6. Neutral emotional engagement. Neutral emotional engagement describes a user’s subdued or indifferent emotional response to content or events.

### Research Questions

User attitudes toward correction may change over time [8], and the misinformation contained in comments is also noteworthy [22]. Therefore, this study first investigated whether user engagement toward health misinformation correction has changed over the past 3 years. Second, according to the FTT, providing the gist as cues can effectively and directly convey messages to users [17]. Accordingly, we hypothesized that providing gist representations would help users grasp the main aspects of health misinformation issues. We also identified some important verbatim representations as post attributes to examine their relationship with user engagement. Finally, we considered whether the content of comments affects users’ choice to share and react to a post. On the basis of the earlier discussion, the research questions (RQs) posed by this study are as follows:

- How has user engagement toward health misinformation correction changed during the COVID-19 pandemic? (RQ1)
- Can the gist and verbatim representations identified in this study as post attributes predict user engagement in shares, reactions, and comments? (RQ2)
- On the basis of RQ2, does the inclusion of user-generated comment content from engagement in commenting affect engagement in sharing and reactions? (RQ3)

## Methods

### Corpus and Sample Period

This study focused on health misinformation correction, drawing on 2 representative platforms, the Taiwan Fact-Checking Center (TFC) and the Ministry of Health and Welfare (MOHW), with rich data, including fact-checking and clarifying health-related event or policy posts and user feedback. Data were collected from the Facebook pages of the TFC, which was established in 2018 and has >160,000 followers on Facebook, and the MOHW, which has a significant following of approximately 1.52 million Facebook users, accounting for 6.45% of the total population of Taiwan. We collected 3769 posts from the TFC and 5103 posts from the MOHW from January 2020 to December 2022. The TFC targets various misinformation issues such as health, politics, economics, and celebrities. By contrast, the post content of MOHW focuses on public health, such as health policy announcements, and the correction of health misinformation accounts for a small portion. To ensure our focus was on health misinformation correction, we manually reviewed each post to identify those that involved both health and misinformation correction. For TFC posts, the reference keywords included health, pandemic, disease, food safety, folk remedies, vaccine, and health care. For MOHW posts, the reference keywords included misinformation, false information, clarification, and fake news. Because relying solely on keywords may either miss or capture non-target content, we primarily relied on manual review. After the filtering process, we found 34.89% (1315/3769) posts from the TFC source and 2.14% (109/5103) posts from the MOHW source. Corresponding to the posts from TFC and MOHW, 26,458 (including 913 self-replies by the TFC manager) and 40,920 comments (including 97 self-replies by the MOHW manager) were downloaded, respectively. Finally, the dataset consisted of 1424 posts and 67,378 comments from both platforms.

### Research Process

#### Overview

**Figure 1:**
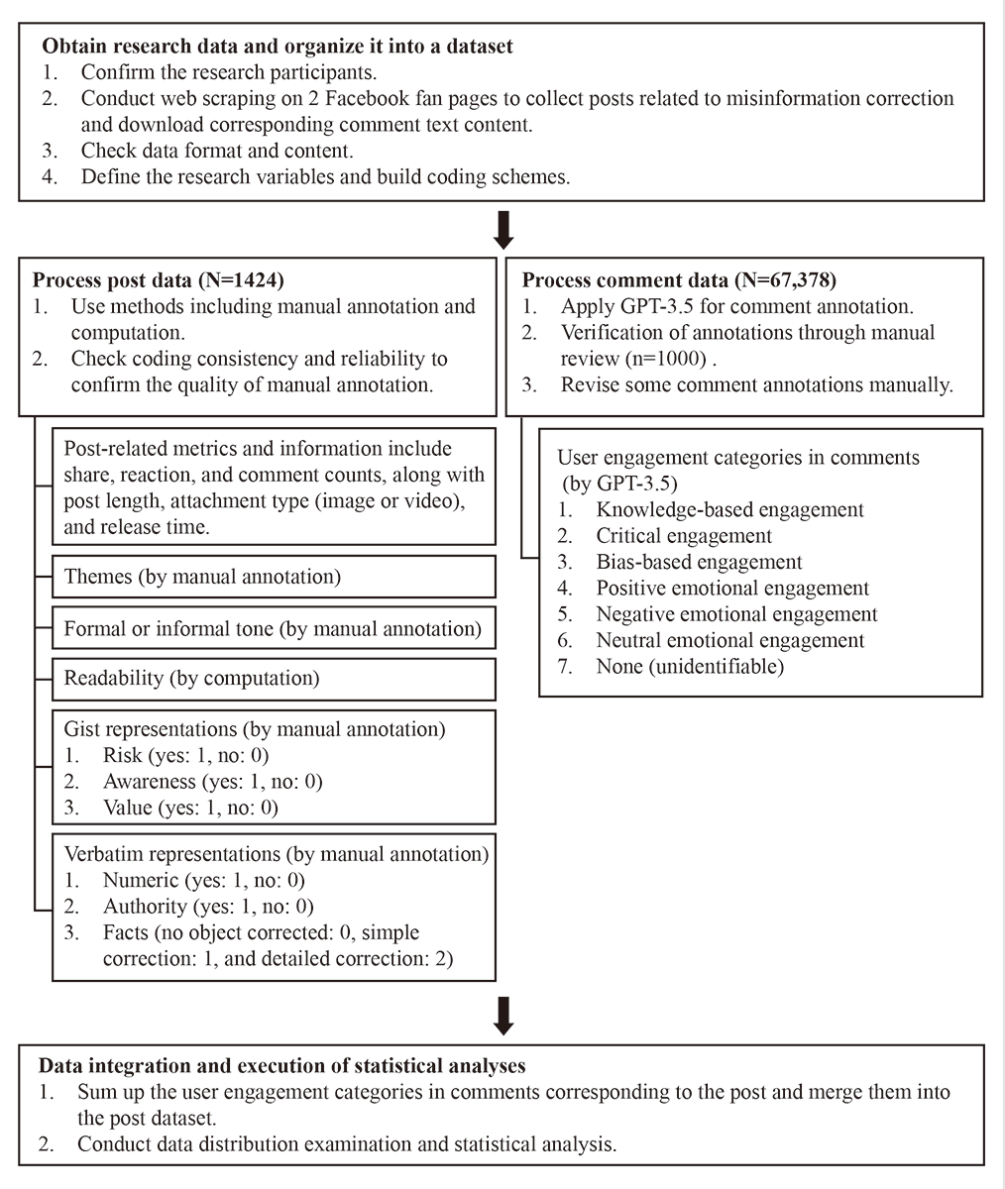
Research procedure and data processing.

This study included three phases, as shown in Figure 1: (1) obtaining research data and organizing them into a dataset, (2) processing post and comment data, and (3) data integration and statistical analyses. First, after defining the research participants, we used web scraping techniques to acquire the necessary posts and relevant metrics and obtain the content of comments. Subsequently, to process unstructured data into structured data for further analysis, manual annotation was applied to posts to obtain 1 indicator (readability) using computational methods, whereas a large amount of comment data was automatically annotated using GPT-3.5 (OpenAI). Finally, a statistical analysis was conducted. Detailed information regarding data annotation processing is provided in the following sections.

#### Annotation and Transformation Data for Posts

Regarding the posts, we categorized them into five main themes based on the emphasized content: (1) understanding and controlling diseases; (2) vaccine safety and policies; (3) health care, food safety, and home remedies; (4) livelihood, economy, and information security; and (5) misinformation awareness and identification. Livelihood, economy, and information security refers to daily life aspects impacted by the COVID-19 pandemic, such as subsidy applications. Two coders manually coded this component (Cohen κ coefficient=0.672). In addition, as control variables, this study considered the length of posts, their readability, whether they were from official platforms, whether they adopted a formal tone, and whether they used images.

Due to the lack of Chinese readability tools, this study created a difficult-word dictionary based on 2 resources: the “Chinese 8000-Word List” levels 3 to 5 [54] and the “National Academy for Educational Research Third Level Seven-Word List” level 7 [55]. In addition, the word frequency table of this dataset was examined, and 340 words, such as reagents and nucleic acid, were added to the dictionary. The difficult-word dictionary included 11,138 words. Then, we used the Dale-Chall readability formula to compute the scores. The formula of the Dale-Chall Readability Score is 0.1579 × [(number of difficult words / total number of words) × 100] + 0.0496 × (total number of words / total number of sentences) [56,57]. The original score ranges from 0 to 10, with higher scores indicating greater difficulty in reading. Furthermore, the additive inverse of this score was calculated for intuitive interpretation, with higher scores indicating easier readability.

Whether a post adopted a formal tone was determined by manual annotation (Fleiss κ=0.521). Because determining whether the tone is formal can be subjective, we gradually reached a consensus throughout the process. This included discussions on the use of common Chinese modal particles and slang. At least 2 annotators annotated each post, and after comparing the annotation results, we discussed and resolved any inconsistencies individually.

The last control variable was whether an image was used. The data we obtained through web scraping included information about attachments, such as images, links, or videos. Uploaded images were prioritized for display in Facebook posts. If no image was uploaded but the content included a link, a preview image was selected from the linked page for display. Similarly, a thumbnail image was shown when a video was uploaded in a post. When an image was used as an attachment, it was typically designed. We did not evaluate the content of the images. Of the 1424 posts, there were 1310 (91.99%) posts with uploaded images as attachments (ranging from 1 to 15 images), 109 (7.65%) posts that displayed an image from a link, 4 (0.28%) posts with an uploaded video, and only 1 (0.07%) post without any attachment. We assigned a dummy code of 1 to the 91.99% (1310/1424) posts with a designed image as an attachment, while the others were coded as 0.

This study focused on examining 6 representations: risk, awareness, value, numeric, authority, and facts, which serve as attributes of misinformation correction posts identified by the FTT and related literature. Please refer to Tables S2 and S3 in Multimedia Appendix 1 for the gist and verbatim coding schemes. This portion of the annotated data was converted into dummy variables. The first author and an annotator reviewed the data during the initial phase and conducted theoretical discussions to create the coding scheme. A 10.04% (143/1424) sample of the posts was manually annotated, and ambiguous concepts were clarified. Two annotators independently annotated another 9.97% (142/1424) of the sample. The interannotator agreement ranged from 87.32% to 94.37%, and Fleiss κ values ranged from 0.459 to 0.927. Each post was coded by at least 2 annotators, and any inconsistencies were discussed and confirmed to reach a consensus.

#### Annotation Data for Comments

Regarding the comments, we classified engagement in comments into 6 categories based on a literature review and empirical data observation. Three categories were related to cognition: knowledge-based, critical, and bias-based engagement. In addition, 3 categories were related to emotion: positive, negative, and neutral. Each comment was assigned 1 of these 6 constructs.

GPT-3.5 has been trained on sufficiently diverse multilingual data and can perform cross-lingual transfer learning by using knowledge from source languages to make inferences in target languages, even with limited target language data [58]. The comment content was mainly in Chinese, and the average length of a comment was 26.40 (SD 52.70) words. We assigned each comment to a category, although it is possible for a single comment to contain multiple categories in short texts. We prioritized using GPT-3.5 to determine cognitive engagement; if not, we assessed for emotional engagement. The comment was classified as None if it was indistinguishable and neither aspect could be determined from the content. Examples of the prompts and outputs are provided in Tables S4 and S5 in Multimedia Appendix 1.

GPT-3.5 has some issues with instability and hallucinations. Regarding annotation instability, we set the temperature parameter to 0.5. This parameter ranges from 0 to 1, with higher values increasing creativity. We found that setting the temperature too low could lead to conservative responses, resulting in frequent annotating as unidentifiable. In the study, approximately 100 comments were used for testing to improve the prompt and adjust the temperature parameter. We required GPT-3.5 to provide reasons during annotation to examine potential causes of annotating errors and incorporated more specific definitions into the prompt. Next, we performed automated annotation of all data. To ensure GPT-3.5 annotation reliability, we randomly selected 1000 comments for manual annotation. Cohen κ coefficient for manual annotations was 0.519 (moderate agreement). After discussion, a consensus on the annotation was reached. The consistency between manual and GPT-3.5 annotations was calculated, yielding a Cohen κ coefficient of 0.648 (substantial agreement). Then, to mitigate potential biases introduced by GPT-3.5, we systematically compared manual annotations with GPT-3.5 annotations and checked the comments for keywords that GPT-3.5 may not recognize. We revised 4233 comment annotations in this process. Finally, the data for the 6 categories of comments were summed and linked to their corresponding posts.

### Data Analysis

We initially used descriptive statistics to provide an overall overview of the data to answer RQ1. The mean share count was 79.12 (SD 293.14; range 0-4259), with 57 posts without sharing. The mean reaction count was 914.35 (SD 2,582.93; range 13-34,403). The mean comment count, excluding self-replies by the platform manager was 46.61 (SD 212.82; range 0-5092), with 198 posts without commenting. The 1.5% (1010/67,378) comments made by the platform manager supplied full information links or expressed positive feedback to some comments. These comments were excluded because we aimed to understand engagement from regular users. After examining the data distribution with Q-Q plots, we found that share, reaction, and comment count were nonnormally distributed. In this situation, it may be appropriate to transform the dependent variable using a natural logarithm in a linear regression model [9,59] or to use Poisson or negative binomial regression models [44,56]. We chose to use natural logarithms of the dependent variables as ln(shares+1), ln(reactions), and ln(comments+1). To improve the interpretability of the model, this study also transformed some independent variables using the natural logarithm, including ln(post length) and ln(engagement categories in comments+1). This study considered post length, readability, formal language use, and images as control variables based on prior literature [56,60]. For the regression models of 3 dependent variables, observations with studentized residuals that fall outside the mean of +3 or –3 were considered outliers and excluded. Then, regression analysis was performed to confirm the theory’s applicability and answer the RQs.

### Ethical Considerations

The study was approved by the National Tsing Hua University Research Ethics Committee (11308HT139). This study used publicly available data and processed and stored the data in compliance with the Personal Data Protection Act. As the data were publicly accessible, informed consent was not required.

## Results

### Overview

To answer RQ1 regarding user engagement trends with misinformation correction during the COVID-19 pandemic, we aggregated 3 years of data and analyzed various types of user engagement. For RQ2, we performed hierarchical multiple regression to examine the relationship between the identified attributes and user engagement in sharing and reactions. For RQ3, we considered the content in comments as additional information on the post, incorporating 6 categories of user engagement in commenting as independent variables in the models for RQ2.

### Trends Related to Post Content and User Engagement During the COVID-19 Pandemic

**Figure 2:**
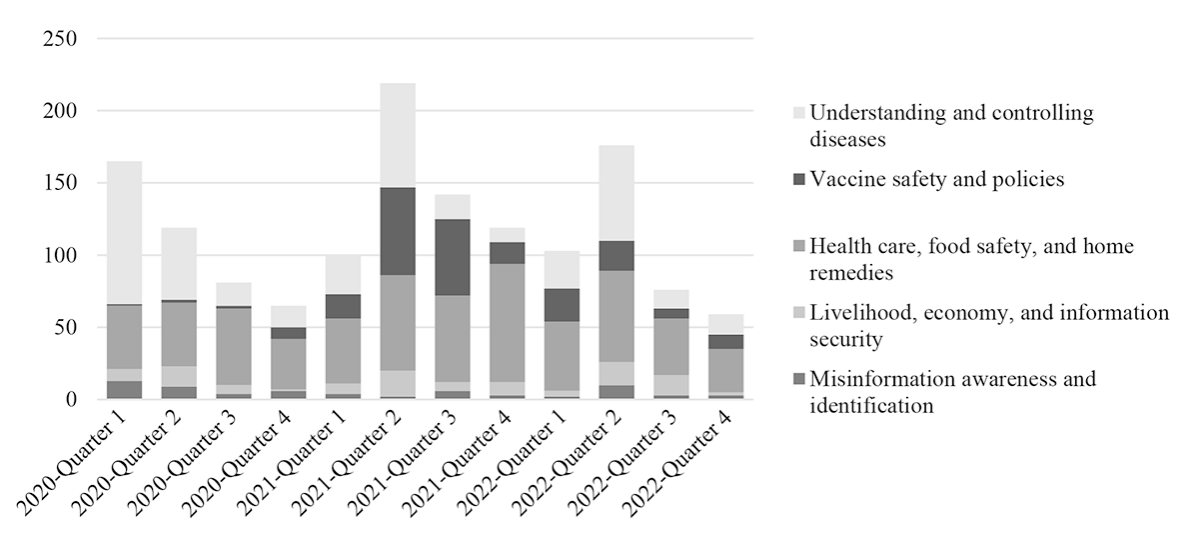
Quarterly distribution of post counts by theme over 3 years.

First, this study organized the data to understand the data profile. We merged the MOHW and TFC datasets to obtain an overview of the situation. We categorized the data by themes based on a quarterly distribution, as shown in Figure 2, in which the distribution of misinformation correction themes aligned with the development trajectory of the COVID-19 pandemic. The peak of misinformation correction occurred in the second quarter of 2021, coinciding with Taiwan’s level 3 alert and a vaccine shortage. The most common theme was health care, food safety, and home remedies, followed by understanding and controlling diseases and vaccine safety and policies.

To answer RQ1, we conducted a 1-way ANOVA to analyze user engagement over 3 years, and the results are shown in Table 1. Through homogeneity tests, these counts were found to be heterogeneous, and Welch ANOVA and Games-Howell post hoc comparisons were performed. We found that sharing (Welch *F*_2, 805.887_=31.082; *P*<.001) and reactions (Welch *F*_2, 802.838_=40.620; *P*<.001) declined significantly over time, while comments (Welch *F*_2, 909.388_=11.253; *P*<.001) remained high in the first 2 years, before dropping in the third year. Under the general trends, we further examined the 3-year change patterns of comments based on user engagement categories. The patterns of the knowledge-based engagement (Welch *F*_2, 931.301_=19.470; *P*<.001) and 3 emotional engagements (positive emotional: Welch *F*_2, 741.713_=22.636; *P*<.001; negative emotional: Welch *F*_2, 845.244_=11.857; *P*<.001; neutral emotional: Welch *F*_2, 849.284_=21.055; *P*<.001) in commenting were similar, remaining consistent in the first and second years but significantly declining in the third year. Critical engagement declined significantly over time (Welch *F*_2, 785.780_=32.996; *P*<.001), while bias-based engagement peaked in the second year and then significantly declined in the third year (Welch *F*_2, 922.113_=3.120; *P*=.045).

**Table 1:** Variation in user engagement over 3 years.

User engagement	2020 (n=430), mean (SD)	2021 (n=580), mean (SD)	2022 (n=414), mean (SD)	Tests of homogeneity of variances		Robust tests of equality of means		Mean difference between groups (Games-Howell post hoc tests)
				Levene statistic	*P* value	Welch statistic	*P* value	
Share	192.65 (496.70)	38.78 (104.67)	17.72 (70.42)	85.546	<.001	31.082	<.001	2020>2021, 2020>2022, 2021>2022
Reaction	1871.12 (3799.06)	664.91 (2001.51)	270.06 (960.27)	60.753	<.001	40.620	<.001	2020>2021, 2020>2022, 2021>2022
Comment	54.28 (131.03)	59.26 (302.90)	20.91 (92.98)	10.115	<.001	11.253	<.001	2020>2022, 2021>2022
Knowledge-based engagement in commenting	7.04 (13.23)	5.42 (22.07)	2.16 (9.98)	11.194	<.001	19.470	<.001	2020>2022, 2021>2022
Critical engagement in commenting	6.80 (13.83)	3.80 (13.32)	1.51 (4.39)	29.049	<.001	32.996	<.001	2020>2021, 2020>2022, 2021>2022
Bias-based engagement in commenting	11.44 (40.04)	25.65 (152.21)	9.00 (44.68)	13.045	<.001	3.120	.045	2021>2022
Positive emotional engagement in commenting	6.87 (20.27)	4.18 (17.30)	1.16 (4.67)	22.438	<.001	22.636	<.001	2020>2022, 2021>2022
Negative emotional engagement in commenting	11.44 (33.40)	11.71 (63.18)	3.67 (17.16)	10.159	<.001	11.857	<.001	2020>2022, 2021>2022
Neutral emotional engagement in commenting	5.00 (11.62)	3.22 (12.38)	1.27 (5.20)	16.948	<.001	21.055	<.001	2020>2022, 2021>2022

**Figure 3:**
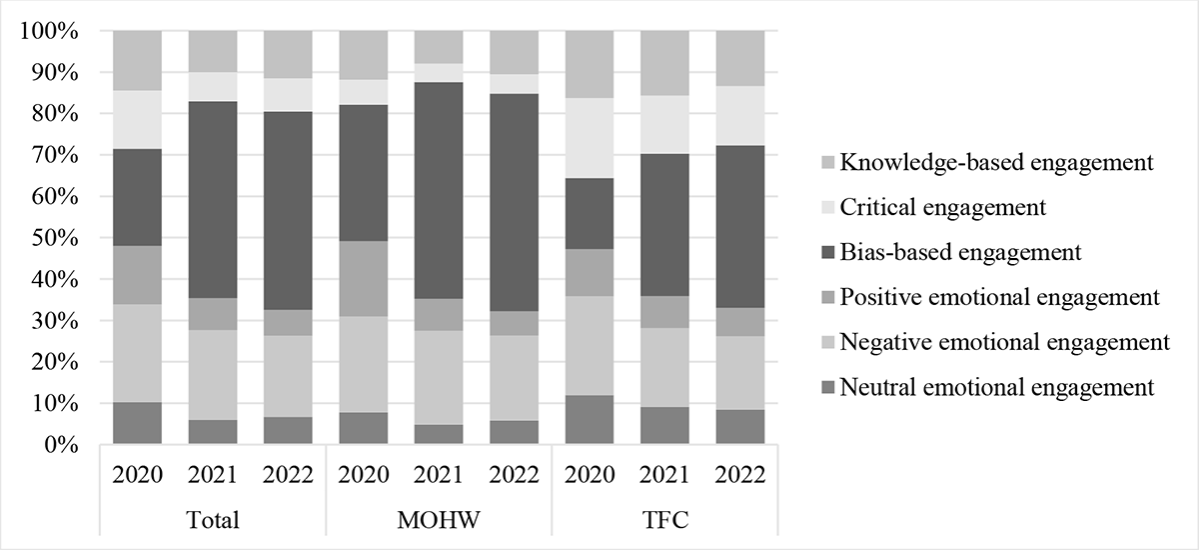
Variation in the proportion of different types of user engagement in comments over 3 years. MOHW: Ministry of Health and Welfare; TFC: Taiwan Fact-Checking Center.

We further explored the relative proportions among different categories of user engagement in commenting, as shown in Figure 3. In the first year of the COVID-19 pandemic, the engagement across categories was fairly balanced, with cognitive and emotional engagement each accounting for approximately half. The sum of knowledge-based and critical engagement comments exceeded those of bias-based engagement in 2020 in overall data, suggesting that the 2 beliefs could counterbalance each other. However, bias-based engagement became predominant by the second and third years on both platforms. In overall data, in 2020, bias-based engagement accounted for 23.55% (4920/20,893) of the comments; in 2021, it accounted for 47.51% (14,876/31,309); and in 2022, it accounted for 47.95% (3726/7770). Specifically, based on the MOHW data, in 2020, bias-based engagement was 32.97% (2776/8419), which rose to 52.39% (11,976/22,858) in 2021 and remained at 52.57% (2662/5064) in 2022. On the TFC data, bias-based engagement was 17.19% (2144/12,474) in 2020, 34.32% (2900/8451) in 2021, and 39.32% (1064/2706) in 2022. The discussions were dominated by bias-based engagement in the later stages of the COVID-19 pandemic. When comparing platforms, there was relatively more knowledge-based and critical engagement on the TFC platform, while the MOHW platform had relatively more positive emotional engagement in the first year.

Objective metrics such as shares, reactions, and comments for user engagement showed that interest in health-related misinformation significantly decreased in the third year. However, regarding the number of deaths due to COVID-19, the pandemic was most severe in Taiwan in its third year, with 7 deaths in 2020, 843 in 2021, and 14,636 in 2022 [61].

### Post Attributes and User Engagement

In the annotated dataset, of the 1424 posts, 261 (18.33%) contained risk, 868 (60.96%) contained awareness, 527 (37.01%) contained value, 163 (11.45%) contained numeric information, and 768 (53.93%) contained authority. In addition, of the 1424 posts, 202 (14.19%) did not target specific misinformation, 505 (35.46%) provided a simple correction, and 717 (50.35%) offered a detailed correction. Subsequently, concerning RQ2, we focused on the relationship between post attributes and 3 user engagement metrics: shares, reactions, and comments, as shown in Table 2. Model 1 controlled for background variables including post length (transformed using the natural logarithm), official status (converted to a dummy variable, where 1 represents the official platform [MOHW] and 0 represents the nonofficial platform [TFC]), readability, formal tone (where 1 indicates formal and 0 indicates informal), and image (where 1 indicates the attachment of at least 1 image and 0 indicates none). The dependent variables were also transformed using the natural logarithm. These background variables contributed significantly to the regression models for shares (*R*^2^=0.334; *F*_5,1412_=141.380; *P*<.001), reactions (*R*^2^=0.407; *F*_5,1404_=192.994; *P*<.001), and comments (*R*^2^=0.328; *F*_5,1414_=137.794; *P*<.001). All background variables were predictive of these engagement metrics, except for post length, which did not significantly predict comment count.

**Table 2:** Hierarchical multiple regression analysis predicting shares, reactions, and comments (models 1 and 2).

Predictors		Share^a^					Reaction^a^					Comment^a^			
		B^b^ (SE^c^)	β^d^	*P* value	VIF^e^	B (SE)		β	*P* value	VIF	B (SE)		β	*P* value	VIF
**Model 1**															
	Constant	3.82 (0.36)	—^g^	<.001	—	6.19 (0.28)		—	<.001	—	1.76 (0.36)		—	<.001	—
	Post length^a^	0.11 (0.05)	.07	.03	2.08	0.11 (0.04)		.08	.005	2.07	0.09 (0.05)		.06	.08	2.09
	Official platform^f^	2.95 (0.14)	.49	<.001	1.15	2.80 (0.11)		.56	<.001	1.15	3.00 (0.14)		.51	<.001	1.15
	Readability	0.39 (0.03)	.27	<.001	1.22	0.30 (0.03)		.25	<.001	1.21	0.16 (0.03)		.11	<.001	1.22
	Formal tone^f^	0.53 (0.11)	.15	<.001	1.95	0.54 (0.08)		.19	<.001	1.94	0.77 (0.10)		.22	<.001	1.95
	Image^f^	0.43 (0.13)	.07	.001	1.09	0.24 (0.10)		.05	.02	1.09	0.27 (0.13)		.05	.04	1.09
**Model 2**															
	Constant	4.40 (0.43)	—	<.001	—	7.00 (0.34)		—	<.001	—	2.56 (0.43)		—	<.001	—
	Post length^a^	–0.04 (0.06)	–.02	.56	3.21	–0.05 (0.05)		–.04	.29	3.20	–0.07 (0.06)		–.04	0.26	3.21
	Official platform	2.88 (0.14)	.48	<.001	1.26	2.84 (0.11)		.57	<.001	1.26	3.11 (0.14)		.52	<.001	1.26
	Readability	0.40 (0.03)	.27	<.001	1.26	0.31 (0.03)		.26	<.001	1.26	0.17 (0.03)		.12	<.001	1.27
	Formal tone^f^	0.45 (0.11)	.13	<.001	2.08	0.42 (0.08)		.15	<.001	2.07	0.63 (0.11)		.18	<.001	2.09
	Image^f^	0.21 (0.14)	.04	.13	1.25	–0.04 (0.11)		–.01	.73	1.26	0.00 (0.14)		.00	.99	1.25
	Risk^f^	0.30 (0.09)	.07	.001	1.11	0.10 (0.07)		.03	.15	1.11	–0.07 (0.09)		–.02	.44	1.11
	Awareness^f^	0.30 (0.07)	.09	<.001	1.14	0.20 (0.06)		.07	.001	1.14	0.21 (0.07)		.06	.005	1.14
	Value^f^	0.14 (0.07)	.04	.051	1.09	0.08 (0.06)		.03	.17	1.08	–0.08 (0.07)		–.02	.28	1.09
	Numeric^f^	0.15 (0.11)	.03	.18	1.10	0.13 (0.09)		.03	.13	1.10	0.27 (0.11)		.06	.02	1.10
	Authority^f^	0.10 (0.10)	.03	.31	2.02	–0.02 (0.08)		–.01	.74	2.04	–0.02 (0.10)		–.01	.84	2.02
	Facts	0.30 (0.08)	.14	<.001	2.71	0.44 (0.06)		.24	<.001	2.73	0.42 (0.08)		.19	<.001	2.72

^a^Transformed using the natural logarithm.

^b^Unstandardized regression coefficient.

^c^SE for unstandardized regression coefficient.

^d^Standardized regression coefficient.

^e^VIF: variance inflation factor.

^f^Converted into a dummy variable.

^g^Not applicable.

Model 2 incorporated gist and verbatim representations. The *R*^2^ changes for the 3 models were significant. In the model focused on predicting shares (*R*^2^=0.353; *F*_11,1406_=69.808; *P*<.001; ∆ *R*^2^=0.020), risk (β=.07; *P*=.001), awareness (β=.09; *P*<.001), and facts (β=.14; *P*<.001) were significant predictors. When predicting reactions (*R*^2^=0.435; *F*_11,1398_=97.292; *P*<.001; ∆ *R*^2^=0.028), awareness (β=.07; *P*=.001) and facts (β=.24; *P*<.001) were found to be significant factors. Similarly, when predicting comments (*R*^2^=0.351; *F*_11,1408_=69.158; *P*<.001; ∆ *R*^2^=0.023), awareness (β=.06; *P*=.005), numeric (β=.06; *P*=.02), and facts (β=.19; *P*<.001) played significant roles. This finding suggests that 2 of the gist representations, risk and awareness, can enhance post endorsement and sharing, but only awareness was predictive of reactions and comments. Moreover, facts positively influenced the 3 metrics in the verbatim dimension, indicating that detailed clarifications enhanced shares, reactions, and comments.

### Post Attributes and User Engagement: Considering Comment Content

For RQ3, we incorporated comments generated by 6 categories of user engagement into model 3, as presented in Table 3. To ensure the validity of our findings, we used the variance inflation factor to diagnose multicollinearity, which was within acceptable limits (<10). In the model predicting shares (*R*^2^=0.668; *F*_17,1400_=165.904; *P*<.001; Δ *R*^2^=0.315), the 3 gist representations (risk: β=.08; *P*<.001, awareness: β=.08; *P*<.001, and value: β=.06; *P*<.001) were significant predictors, while facts (β=.02; *P*=.36) no longer predicted it. Then, when considering user engagement in commenting, in the cognitive aspect, only bias-based engagement (β=–.11; *P*=001) negatively predicted shares, and knowledge-based and critical engagement positively predicted shares (knowledge-based: β=.28; *P*<.001 and critical: β=.15; *P*<.001). In the emotional aspect, positive and negative emotional engagement both predicted shares, while neutral did not predict it (positive: β=.29; *P*<.001, negative: β=.09; *P*=.02, and neutral: β=.05; *P*=.15). Similarly, in the model predicting reactions (*R*^2^=0.772; *F*_17,1392_=277.743; *P*<.001; Δ *R*^2^=0.337), the 3 gist representations (risk: β=.04; *P*=.007, awareness: β=.06; *P*<.001, and value: β=.05; *P*<.001) significantly predicted the reaction count, and facts (β=.12; *P*<.001) also predicted it. All comment categories, except for bias-based engagement (β=–.04; *P*=.15), significantly predicted reactions (knowledge-based: β=.19; *P*<.001, critical: β=.14; *P*<.001, positive: β=.26; *P*<.001, negative: β=.15; *P*=<.001, and neutral: β=.08; *P*=.002).

**Table 3:** Hierarchical multiple regression analysis predicting share and reaction count (model 3).

Predictors			Share^a^								Reaction^a^						
			B^b^ (SE^c^)		β^d^		*P* value		VIF^e^		B (SE)		β		*P* value		VIF
**Model 3**																	
	Constant	2.34 (0.32)		—^f^		<.001				5.26 (0.22)		—		<.001		—	
	Post length^a^	0.08 (0.05)		.05		.07		3.27		0.05 (0.03)		.03		.13		3.27	
	Official platform^g^	0.72 (0.14)		.12		<.001		2.18		0.91 (0.09)		.18		<.001		2.18	
	Readability	0.24 (0.03)		.17		<.001		1.32		0.18 (0.02)		.15		<.001		1.31	
	Formal tone^g^	0.02 (0.08)		.01		.76		2.14		0.03 (0.05)		.01		.58		2.13	
	Image^g^	0.23 (0.10)		.04		.02		1.25		–0.02 (0.07)		.00		.79		1.26	
	Risk^g^	0.31 (0.07)		.08		<.001		1.11		0.12 (0.05)		.04		.007		1.11	
	Awareness^g^	0.25 (0.05)		.08		<.001		1.16		0.15 (0.04)		.06		<.001		1.15	
	Value^g^	0.19 (0.05)		.06		<.001		1.11		0.15 (0.04)		.05		<.001		1.10	
	Numeric^g^	0.01 (0.08)		.00		.92		1.11		0.00 (0.06)		.00		.93		1.11	
	Authority^g^	0.07 (0.07)		.02		.28		2.02		–0.04 (0.05)		–.01		.46		2.05	
	Facts	0.05 (0.06)		.02		.36		2.78		0.22 (0.04)		.12		<.001		2.80	
	Knowledge-based engagement^a^	0.42 (0.04)		.28		<.001		3.67		0.24 (0.03)		.19		<.001		3.68	
	Critical engagement^a^	0.23 (0.05)		.15		<.001		3.77		0.18 (0.03)		.14		<.001		3.78	
	Bias-based engagement^a^	–0.13 (0.04)		–.11		.001		4.29		–0.04 (0.03)		–.04		.15		4.26	
	Positive emotional engagement^a^	0.45 (0.05)		.29		<.001		4.36		0.34 (0.03)		.26		<.001		4.35	
	Negative emotional engagement^a^	0.12 (0.05)		.09		.02		6.93		0.17 (0.04)		.15		<.001		6.84	
	Neutral emotional engagement^a^	0.08 (0.05)		.05		.15		4.26		0.11 (0.04)		.08		.002		4.27	

^a^Transformed using the natural logarithm.

^b^Unstandardized regression coefficient.

^c^SE for unstandardized regression coefficient.

^d^Standardized regression coefficient.

^e^VIF: variance inflation factor.

^f^Not applicable.

^g^Converted into a dummy variable.

## Discussion

### Principal Findings

First, in response to RQ1, all metrics unsurprisingly decreased, and it is noteworthy that the proportion of bias-based engagement in comments increased on both platforms. At the outbreak of the COVID-19 pandemic, despite the potential chaos in information, the comment categories remained relatively balanced and were not dominated by any category. The increase in bias-based engagement in the second year was likely due to the first COVID-19 community transmission in Taiwan and the rise of COVID-19 vaccine–related issues. The result aligns with findings from previous studies, which show that comments often contain inaccuracies and are predominantly made by uninformed users [22,52].

Then, we compared our findings with similar studies that explored the relationship between post attributes and user engagement. Regarding background variables, according to model 1, readability aligned with previous research findings [56], which explained why readability influences social media engagement through fluency. Posts using a formal tone also showed higher user engagement, consistent with the literature [60], in which formality was a heuristic for credibility and importance. However, we did not control for the topics or timing associated with using a formal tone; more severe events may be more likely to use a formal tone. Posts containing images also positively impacted user engagement, a finding supported and explained in the literature by the concept of media type richness [59] or an approach-motivated perspective [44].

Drawing on the FTT and literature on misinformation, we identified 3 key gist representations and tested their effectiveness. The theory suggests that a message with a clear core and causal explanation is more likely to resonate with people, leading to behaviors such as sharing [30]. Moreover, this study suggests that gist representations provide cues that enhance the probability of users finding the respective information relatable or practical. Risk may involve terms such as danger or harm. Awareness often describes tips and tricks and is relatively neutral. Value sometimes refers to reassurance and tends to have more positive terms. However, only awareness is a significant predictor in each model. When considering the content of comments, all gist representations significantly predict reactions, indicating that risk and value retain their value within social media posts that include comments. In addition, value was the only gist of predicting comments negatively, although not significantly. Whether value can further reduce bias-based engagement remains to be examined in future studies. Overall, providing gist information increased shares, reactions, and comments, consistent with previous research showing that gist information enhances article sharing [14]. Awareness of misinformation is the most crucial factor in this study, as a previous study has emphasized awareness of misinformation as the foundational concept of citizen resilience to misinformation [35].

Regarding the verbatim dimension, we found that numeric information cannot significantly predict shares and reactions but can predict comments. Statistics also failed to predict sharing in another study based on the FTT [14]. According to discussions related to the FTT, individuals’ numerical abilities influence the numeric factor [38], and users might engage in discussions based on different perceptions of numeric information. However, statistics as a factor of source transparency was one of the predictors of shares and comments, suggesting that numerical information in news articles may increase the audience’s perceived accuracy [9]. A previous study indicated that participants engaged more with corrections even without statistical information due to the simplicity of the material, which made it easier to assess credibility. Then, they put less cognitive effort when the information included statistical evidence [20]. We offered a different perspective through FTT, suggesting that numeric information did not significantly affect user engagement in sharing and reacting, as these forms of engagement relied more on top-down meaning. However, numeric information increased commenting, which might rely on detailed information and higher cognitive engagement. Furthermore, this study identified authority as verbatim because the names of experts or institutes as references were detailed. Nevertheless, the attribute still conveyed a sense of credibility. The concept was similar to source transparency but with slight differences, and a previous study showed that using official reports increased shares and comments, and references to laws and news articles also increased comments [9]. In this study, this variable did not significantly predict any metrics. This could also be due to both platforms being trustworthy sources. Finally, facts significantly predicted all 3 metrics in model 2, indicating that posts with detailed and complete content generated higher user engagement, which is consistent with past experimental results [12,13,18]. Detailed corrections might contain alternative interpretations and context information for understanding and discussion, generating user engagement.

Finally, we considered that readers might refer to comment content to decide whether to share or react in model 3. An increase in knowledge-based and critical engagement comments, which reflect scientific beliefs, led to higher shares and reactions. Conversely, comments reflecting bias-based engagement negatively predicted shares, suggesting that these comments, much like uncivil comments [47], may diminish the quality of discussion threads and reduce share counts. Comments with positive and negative emotional engagement both predicted shares and reactions. Although comment categories could predict shares and reactions, all 3 gist representations still play an important role, indicating that gist communication can still be effective.

In summary, this study defined post attributes based on the FTT and considered a more diversified array of user engagement types to explore their relationship with post attributes. The main theoretical contribution of our study is that it presents a framework applied to misinformation issues validated using real social media data. It examined user engagement in commenting to better reflect the content users are most likely to encounter. The main practical contribution is the introduction of gist representations—risk, awareness, and value—that serve as simple but important cues when publishing correction messages, helping users grasp and address misinformation issues and encouraging content sharing.

### Implications, Limitations, and Future Directions

FTT emphasizes the mutually supportive relationship between our cognition and social values and recommends using reasoned approaches to guide decision-making and emotional responses, rather than emotional appeals, to counter misinformation [17]. Building on this foundation, we identified 3 rational gist representations that connect with social values. Among these, awareness of misinformation proved to be a highly effective gist when comment content was not considered. In our data, this concept included elements such as increasing awareness of misinformation and fraud, encouraging verification of sources, promoting cautious sharing, and identifying characteristics and sources of misinformation. Mentioning the risks associated with misinformation can increase share counts. The data for this study referenced health risks, social panic, legal risks, and information security risks. Mentioning the value of health promotion is effective when considering comment content. The data for this study referenced health promotion and health literacy, a positive attitude toward the COVID-19 pandemic prevention, media or eHealth literacy, public trust, psychological well-being, and social confidence. These attributes serve as guidelines for designing content in misinformation correction and science communication.

This study had some limitations. First, this study examined data from 3 years during the COVID-19 pandemic, during which attention to health misinformation was heightened. There were also some health crises before and after the COVID-19 pandemic. Whether inferences based on the results of this study remain valid requires further examination. Second, we used GPT-3.5 to code comments efficiently, followed by a manual review. Cultural characteristics may have caused GPT-3.5 to misinterpret specific comments. For example, the colors blue and green sometimes represent political parties, and GPT-3.5 may not always accurately interpret this from the context, leading to some classification inaccuracies. Nevertheless, GPT-3.5 offers several advantages over previous machine learning methods, such as cross-language capabilities and emoji interpretation. Third, authority, as one of the independent variables in this study, has implications for credibility in some types of research. In the heuristic-systematic model, it is viewed as having an intuitive role, and in the elaboration likelihood model, it is considered a peripheral cue, suggesting the credibility of a message. We cannot confirm whether the dual roles cause construct confusion or whether the platform symbolizes knowledge authority. Authority did not significantly predict the outcomes in models 2 and 3; however, this does not imply that it is unimportant. Finally, we considered whether there was an attached image but did not examine its content or whether the images contained gist messages. Images attract user attention more quickly and should be considered in future research.

In addition to addressing the abovementioned limitations, future research could consider simplifying the 6 comment categories we used. For instance, knowledge-based and critical engagement could be combined, as well as positive and neutral emotional engagement. In addition, some comments directly responded to the content design of certain corrections. We observed that some posts were criticized for lacking focus, while others were praised for being practical. If these comments can be accurately extracted and systematically analyzed, it would also help assess whether this type of gist communication is well received. We found that posts containing numerical information, such as vaccine adverse reaction rates, received more comments, as users may interpret and engage more with rates and numbers. This area requires more systematic analysis and effective framing of numeric information in correction design to prevent misinterpretation on social media, enhancing the application of FTT. Finally, this study used regression analysis to explore the impact of 6 attributes on metrics; however, a single post often contains multiple attributes, and it may be possible to identify an optimal combination. Future studies could further explore this area.

### Conclusions

This study enriches the theoretical understanding of the relationship between user engagement and the effectiveness of web-based communication strategies in conveying corrections for health misinformation. It provides evidence-based web content for delivering health misinformation corrections, and FTT explains user engagement with specific post attributes. The theoretical contribution strengthens the link between FTT and multiple engagement metrics beyond sharing and discusses post attributes in terms of gist and verbatim dimensions. In addition, by incorporating comment content, gist communication increases shares and reactions effectively. Finally, these findings provide a foundation for designing more effective content strategies to combat health misinformation, ultimately contributing to more resilient public health communication.

## Appendix

Multimedia Appendix 1 Examples of health misinformation correction, coding schemes, prompts for GPT-3.5, and generated output examples.

## Abbreviations

FTT: fuzzy-trace theory
MOHW: Ministry of Health and Welfare
RQ: research question
TFC: Taiwan Fact-Checking Center

## Data Availability

The datasets generated during and analyzed during this study are available from the corresponding author on reasonable request.

## References

[1] Ecker UK, Lewandowsky S, Cook J, Schmid P, Fazio LK, Brashier N, Kendeou P, Vraga EK, Amazeen MA (2022). The psychological drivers of misinformation belief and its resistance to correction. Nat Rev Psychol.

[2] Gallotti R, Valle F, Castaldo N, Sacco P, De Domenico M (2020). Assessing the risks of 'infodemics' in response to COVID-19 epidemics. Nat Hum Behav.

[3] Cinelli M, De Francisci Morales G, Galeazzi A, Quattrociocchi W, Starnini M (2021). The echo chamber effect on social media. Proc Natl Acad Sci U S A.

[4] Domenico GD, Sit J, Ishizaka A, Nunan D (2021). Fake news, social media and marketing: a systematic review. J Bus Res.

[5] Yoon HY, You KH, Kwon JH, Kim JS, Rha SY, Chang YJ, Lee S (2022). Understanding the social mechanism of cancer misinformation spread on YouTube and lessons learned: infodemiological study. J Med Internet Res.

[6] Bode L, Vraga EK (2018). See something, say something: correction of global health misinformation on social media. Health Commun.

[7] Chu WM, Shieh GJ, Wu SL, Sheu WH (2020). Use of Facebook by academic medical centers in Taiwan during the COVID-19 pandemic: observational study. J Med Internet Res.

[8] Xue H, Gong X, Stevens H (2022). COVID-19 vaccine fact-checking posts on Facebook: observational study. J Med Internet Res.

[9] Kim HS, Suh YJ, Kim EM, Chong E, Hong H, Song B, Ko Y, Choi JS (2022). Fact-checking and audience engagement: a study of content analysis and audience behavioral data of fact-checking coverage from news media. Digital Journalism.

[10] Lewandowsky S, Ecker UK, Seifert CM, Schwarz N, Cook J (2012). Misinformation and its correction: continued influence and successful debiasing. Psychol Sci Public Interest.

[11] Koh HK, Rudd RE (2015). The arc of health literacy. JAMA.

[12] van der Meer TG, Jin Y (2020). Seeking formula for misinformation treatment in public health crises: the effects of corrective information type and source. Health Commun.

[13] Zeng HK, Lo SY, Li SS (2024). Credibility of misinformation source moderates the effectiveness of corrective messages on social media. Public Underst Sci.

[14] Broniatowski DA, Hilyard KM, Dredze M (2016). Effective vaccine communication during the disneyland measles outbreak. Vaccine.

[15] Reyna VF, Brainerd CJ (1995). Fuzzy-trace theory: an interim synthesis. Learn Individ Differ.

[16] Blalock SJ, DeVellis RF, Chewning B, Sleath BL, Reyna VF (2016). Gist and verbatim communication concerning medication risks/benefits. Patient Educ Couns.

[17] Reyna VF (2021). A scientific theory of gist communication and misinformation resistance, with implications for health, education, and policy. Proc Natl Acad Sci U S A.

[18] Martel C, Mosleh M, Rand DG (2021). You’re definitely wrong, maybe: correction style has minimal effect on corrections of misinformation online. Media Commun.

[19] Wang Y (2021). Debunking misinformation about genetically modified food safety on social media: can heuristic cues mitigate biased assimilation?. Sci Commun.

[20] Song Y, Wang S, Xu Q (2022). Fighting misinformation on social media: effects of evidence type and presentation mode. Health Educ Res.

[21] Sui Y, Zhang B (2021). Determinants of the perceived credibility of rebuttals concerning health misinformation. Int J Environ Res Public Health.

[22] Bizzotto N, Schulz PJ, de Bruijn GJ (2023). The "Loci" of misinformation and its correction in peer- and expert-led online communities for mental health: content analysis. J Med Internet Res.

[23] McGuire WJ (1961). Resistance to persuasion conferred by active and passive prior refutation of the same and alternative counterarguments. J Abnorm Soc Psychol.

[24] Petty RE, Cacioppo JT, Berkowitz L (1986). The elaboration likelihood model of persuasion. Advances in Experimental Social Psychology.

[25] Chaiken S (1980). Heuristic versus systematic information processing and the use of source versus message cues in persuasion. J Pers Soc Psychol.

[26] Edelson SM, Reyna VF (2024). Who makes the decision, how, and why: a fuzzy-trace theory approach. Med Decis Making.

[27] Broniatowski DA, Reyna VF (2020). To illuminate and motivate: a fuzzy-trace model of the spread of information online. Comput Math Organ Theory.

[28] Reyna VF, Brainerd CJ (2011). Dual processes in decision making and developmental neuroscience: a fuzzy-trace model. Dev Rev.

[29] Reyna VF, Edelson S, Hayes B, Garavito D (2022). Supporting health and medical decision making: findings and insights from fuzzy-trace theory. Med Decis Making.

[30] Broniatowski DA, Hosseini P, Porter EV, Wood TJ (2024). The role of mental representation in sharing misinformation online. J Exp Psychol Appl.

[31] Ecker UK, O'Reilly Z, Reid JS, Chang EP (2020). The effectiveness of short-format refutational fact-checks. Br J Psychol.

[32] Blalock SJ, Reyna VF (2016). Using fuzzy-trace theory to understand and improve health judgments, decisions, and behaviors: a literature review. Health Psychol.

[33] Reyna VF (2012). Risk perception and communication in vaccination decisions: a fuzzy-trace theory approach. Vaccine.

[34] Dryhurst S, Schneider CR, Kerr J, Freeman AL, Recchia G, van der Bles AM, Spiegelhalter D, van der Linden S (2020). Risk perceptions of COVID-19 around the world. J Risk Res.

[35] Rodríguez-Pérez C, Canel MJ (2023). Exploring European citizens’ resilience to misinformation: media legitimacy and media trust as predictive variables. Media Commun.

[36] Rimal RN, Lapinski MK (2009). Why health communication is important in public health. Bull World Health Organ.

[37] Pan W, Liu D, Fang J (2021). An examination of factors contributing to the acceptance of online health misinformation. Front Psychol.

[38] Reyna VF, Brainerd CJ (2023). Numeracy, gist, literal thinking and the value of nothing in decision making. Nat Rev Psychol.

[39] Looi JC, Allison S, Bastiampillai T, Maguire PA (2021). Clinical update on managing media exposure and misinformation during COVID-19: recommendations for governments and healthcare professionals. Australas Psychiatry.

[40] Bautista JR, Zhang Y, Gwizdka J (2021). Healthcare professionals' acts of correcting health misinformation on social media. Int J Med Inform.

[41] Chan MP, Jones CR, Hall Jamieson K, Albarracín D (2017). Debunking: a meta-analysis of the psychological efficacy of messages countering misinformation. Psychol Sci.

[42] Moorhead SA, Hazlett DE, Harrison L, Carroll JK, Irwin A, Hoving C (2013). A new dimension of health care: systematic review of the uses, benefits, and limitations of social media for health communication. J Med Internet Res.

[43] Kim C, Yang SU (2017). Like, comment, and share on Facebook: how each behavior differs from the other. Public Relat Rev.

[44] Rus HM, Cameron LD (2016). Health communication in social media: message features predicting user engagement on diabetes-related Facebook pages. Ann Behav Med.

[45] Jiang S, Tay J, Ngien A, Basnyat I (2024). Social media health promotion and audience engagement: the roles of information dissemination, organization-audience interaction, and action confidence building. Health Commun.

[46] Bhattacharya S, Srinivasan P, Polgreen P (2017). Social media engagement analysis of U.S. Federal health agencies on Facebook. BMC Med Inform Decis Mak.

[47] Weber P, Prochazka F, Schweiger W (2019). Why user comments affect the perceived quality of journalistic content: the role of judgment processes. J Media Psychol.

[48] Mehmet M, Heffernan T, Algie J, Forouhandeh B (2021). Harnessing social listening to explore consumer cognitive bias: implications for upstream social marketing. J Soc Mark.

[49] Brugnoli E, Cinelli M, Quattrociocchi W, Scala A (2019). Recursive patterns in online echo chambers. Sci Rep.

[50] Colliander J (2019). “This is fake news”: investigating the role of conformity to other users’ views when commenting on and spreading disinformation in social media. Comput Human Behav.

[51] Hong S, Cameron GT (2017). Will comments change your opinion? The persuasion effects of online comments and heuristic cues in crisis communication. J Conting Crisis Manag.

[52] Bode L, Vraga EK (2021). Correction experiences on social media during COVID-19. Soc Media Soc.

[53] Russell JA, Barrett LF (1999). Core affect, prototypical emotional episodes, and other things called emotion: dissecting the elephant. J Pers Soc Psychol.

[54] Chinese 8,000-word list. National Chinese Quiz Promotion Working Committee.

[55] Third level seven-word list. National Academy for Educational Research.

[56] Pancer E, Chandler V, Poole M, Noseworthy TJ (2018). How readability shapes social media engagement. J Consum Psychol.

[57] Dale E, Chall JS (1948). A formula for predicting readability: instructions. Educ Res Bull.

[58] Feng K, Huang L, Wang K, Wei W, Zhang R (2024). Prompt-based learning framework for zero-shot cross-lingual text classification. Eng Appl Artif Intell.

[59] Sabate F, Berbegal-Mirabent J, Cañabate A, Lebherz PR (2014). Factors influencing popularity of branded content in Facebook fan pages. Eur Manag J.

[60] Linos E, Lasky-Fink J, Larkin C, Moore L, Kirkman E (2024). The formality effect. Nat Hum Behav.

[61] Statistics table of area, age, sex of death cases - COVID-19 (by month of death). Center for Diseases Control, Ministry of Health and Welfare.

