# Supplementary material for: Predicting User Engagement in Health Misinformation Correction on Social Media Platforms in Taiwan: Content Analysis and Text Mining Study
Source: J Med Internet Res. 2025 Jan 23;27:e65631. doi: 10.2196/65631 (PMC11803327; doi:10.2196/65631)
Supplement: Multimedia Appendix 1 [file jmir_v27i1e65631_app1.docx]

## Multimedia Appendix: Supplemental Material

Table S1. Formats and examples of health misinformation correction in this study.

| Formats of Misinformation Correction | Example |
| --- | --- |
| 1. Clarifying health misinformation | #Clarification Time By MOHW #Please Help Share This Clarification #Even Conan Said It Cyanide is highly toxic and can be fatal, do not use it to prevent the novel coronavirus #President Shiba Is Furious  Recently, some netizens spread comments such as "Cyanide can kill the Wuhan pneumonia virus and prevent infection, Chinese people should take it quickly..." on social networking sites...  The CECC severely denounced today (8th) that cyanide is highly toxic. If someone mistakenly believes this statement and takes it, it will endanger their or others' health. It also emphasized that the coronavirus has an envelope, and it is straightforward to use alcohol or bleach. It is easy to disinfect effectively, so we urge the public not to forward messages from unknown sources at will to avoid breaking the law.  The CECC pointed out that cyanide can damage the brain and heart, causing unconsciousness, cramps, coma, shock, and even death. The public is advised not to believe or try any epidemic prevention information or remedies from unknown sources to ensure their safety and that of others.  #Regarding Rumors  We sincerely ask everyone to share this information. Maybe some rumors are absurd to some people, but everyone is unique, has different majors, and spends time focusing on other things in life. We even believe things that others find ridiculous at some point! Let's work together to spread accurate information and let more people know! (bows)  Original text (2020-02-08 16:58:26):  #衛福編編澄清時間 #幫衛福編編分享拜託 #柯南也講過  氰化物為劇毒且可能致命，勿拿來防新型冠狀病毒 #總柴震怒  近日有網友於社群網站散布「氰化物可殺死武漢肺炎病毒跟預防染病 中國人快點服用….」等言論......  中央流行疫情指揮中心今(8)日嚴斥，氰化物屬劇毒，若有人誤信該言論而服用，將危害自身或他人健康，並強調冠狀病毒具外套膜，使用酒精、漂白水就很容易有效消毒，因此呼籲民眾勿隨意轉發來路不明訊息，以免觸法。  指揮中心指出，氰化物會傷大腦、心臟，導致意識不清，抽筋、昏迷、休克，甚至死亡。請民眾收到來路不明的防疫資訊或偏方勿輕易相信或嘗試，以確保自身及他人安全。  #關於謠言  編編懇請大家分享，或許有的謠言對一些人來說很扯，但每個人都是特別的，有不同的專業，人生也花時間關注不同的事，編編也曾經對一些人家覺得很扯的事深信不疑過呢～  我們一起把正確資訊分享出去，讓更多人知道吧！（鞠躬） |
| 2. Fact-checking includes the process of verifying information | [False] It is said on the Internet, "Don't worry if you can't buy a mask. 15 masks are enough. Take out the first one on the 15th day and use it. The virus has been isolated for 14 days."?  It is said on the Internet, "Don't worry if you can't buy a mask. 15 masks are enough. Take out the first one on the 15th day and use it. The virus has been isolated for 14 days." After investigation:  1. The "14 days" mentioned in the epidemic prevention measures refer to the 14-day incubation period for the virus to enter the human body and is not the time for the virus to survive on the surface of the mask.  2. Experts point out that bacteria will breed in used masks if left for 14 days. The rumored reuse method is not feasible.  Therefore, the rumor is false information.  Background  A message on the social platform pointed out: "Don't worry if you can't buy a mask. 15 masks are enough. Take out the first one on the 15th day and use it. The virus has been isolated for 14 days."  Fact-Check  Controversial point: Can masks be reused after 14 days?  The TFC interviewed Xie Si-min, an attending physician at the Department of Infectious Diseases at the National Taiwan University Hospital. He pointed out that people who come into contact with possible sources of infection must be quarantined for 14 days. This means that the virus may have a 14-day incubation period in the human body. If no symptoms occur after 14 days of observation, the virus may not have been infected. Infect. Therefore, "14 days" is not the time for the virus to survive on the surface of the mask.  Xie Si-min said that if the mask is contaminated with the virus after use, there is no point in keeping it for 14 days. Moreover, after the mask is used, the inner layer of the mask will be contaminated with mouth droplets, and bacteria will breed if left for 14 days. Therefore, it is not recommended that people keep the mask for 14 days before reusing it.  The TFC interviewed Huang Shi-ze, a chronic infectious disease prevention physician from the Department of Disease Control and Prevention of the Ministry of Health and Welfare. Huang Shi-ze pointed out that "mask use" and "virus disappearance" differ. The protective effect of the mask will become worse after use. Therefore, it is not recommended that the mask be reused.  Conclusion  1. The "14 days" mentioned in the epidemic prevention measures refer to the 14-day incubation period for the virus to enter the human body and is not the time for the virus to survive on the surface of the mask.  2. Experts point out that bacteria will breed in used masks if left for 14 days. The rumored reuse method is not feasible.  Therefore, the rumor is false information.  For the complete fact-check report, please visit the TFC website.  Original text (2020-02-19 08:30:20):  【錯誤】網傳「買不到口罩別急，有15個口罩就夠用了，到第15天再把第一個拿出來用，病毒都隔離14天了」？  網傳「買不到口罩別急，有15個口罩就夠用了，到第15天再把第一個拿出來用，病毒都隔離14天了」，經查：  一、防疫措施所提及的「14天」，是指病毒到人體內可能有14天的潛伏期，並不是病毒能在口罩表面存活的時間。  二、專家指出，用過的口罩放14天會有細菌孳生，傳言所提的重複使用方式，並不可行。  因此，傳言為「錯誤」訊息。  背景  社群平台流傳訊息指出：「買不到口罩別急，有15個口罩就夠用了，到第15天再把第一個拿出來用，病毒都隔離14天了。」  查核  爭議點、用過的口罩可以放14天後重複使用嗎？  查核中心採訪台大醫院感染科主治醫師謝思民，他指出，接觸到可能的傳染源要隔離14天，是指病毒到人體內可能有14天的潛伏期，如果觀察14天後沒有發病，就代表可能沒有得到感染。所以「14天」不是病毒在口罩表面存活的時間。  謝思民說，如果口罩經過使用沾染到病毒，放14天是沒有意義的。而且口罩經過使用，口罩內層會沾染到口沫，放14天會有細菌孳生，因此不建議民眾把口罩放14天再重複使用。  查核中心採訪衛生福利部疾病管制署慢性傳染病防疫醫師黃士澤，黃士澤指出，「口罩使用」與「病毒消失」是兩件事情，口罩使用過後保護效果就會變差，因此，不建議口罩如此重複使用。  結論  一、防疫措施所提及的「14天」，是指病毒到人體內可能有14天的潛伏期，並不是病毒能在口罩表面能存活的時間。  二、專家指出，用過的口罩放14天會有細菌孳生，傳言所提的重複使用方式，並不可行。  因此，傳言為「錯誤」訊息。  完整查核報告請見台灣事實查核中心網站 |
| 3. Addressing misinformation without directly correcting a specific claim | Research and Trends: "Mastering the Vaccine Misinformation Playbook" - The Fact-Check Center examines the patterns of vaccine rumors.  For the vaccine epidemic prevention war to be successful, in addition to the medical technology of the vaccine itself, the entire society must also obtain accurate and sufficient vaccine information not to be affected by misinformation and conspiracy theories and sway their willingness to get vaccinated.  It is worth noting that as the vaccine development progresses, misinformation appearing in various countries worldwide has invariably shown similarities. In mid-May this year, the TFC sorted out the routines of vaccine rumors and made advance arrangements for the infodemic.  Original text (2021-09-16):  #研究與動態 〈掌握疫苗的 #不實訊息 劇本 查核中心整理出疫苗謠言的套路〉  疫苗防疫戰要能夠成功，除了疫苗本身的醫學技術，整個社會民眾也必須獲得準確而充分的疫苗資訊，才不會被不實訊息與陰謀論影響，動搖接種意願。  值得關注的是，隨著疫苗發展進程，世界各國出現的不實訊息，不約而同展現了相似性。今年5月中旬，查核中心整理出疫苗謠言的套路，為謠言疫情超前部署。 |

Table S2. Gist coding scheme.

| **Gist coding scheme** | **Aspects covered in the data** | **Description** | **Example** |
| --- | --- | --- | --- |
| **Risks** associated with misinformation yes: 1, no: 0 | Health risks | Promoting ineffective or harmful remedies and medical advice harms personal health or delays treatment. | "Applying certain substances for a long time is harmful to the body, not recommended." "Erythromycin cannot treat COVID-19, abuse may lead to antibiotic resistance risk." "If people self-medicate, it may delay seeking medical treatment." |
|  | Social panic | Explains how spreading misinformation or exaggerating the severity of diseases can cause public panic. | "This message ignites panic and anxiety in many people." "These false rumors can cause unnecessary panic and tension." |
|  | Legal risks | Mentions the legal consequences of spreading misinformation. | "Avoid violating the Infectious Disease Control Law." "Legally, a maximum fine of 3 million or imprisonment of up to 3 years." |
|  | Information security risks | Spreading links with malware or fraud messages under the guise of pandemic prevention or subsidies poses a risk to information security. | "Clicking on unknown links may lead to malicious software stealing personal information." |
| **Awareness** of misinformation yes: 1, no: 0 | Increased awareness of misinformation and fraud | Reminds the public to stay vigilant against potential misinformation and fraud messages. | "Stop believing in baseless dietary remedies." |
|  | Encouraging source verification | Encourages and reminds people to check and verify the sources of information. | "If in doubt, use the Criminal Police Bureau's 165 anti-fraud hotline for verification." |
|  | Sharing information with caution | Emphasizes careful consideration before sharing any information to avoid spreading potential misinformation. | "Pay attention to and verify unverified messages; do not forward or spread." "Such messages are hard to verify as true or false and can easily cause panic, so it is advised not to forward them." |
|  | Identifying characteristics and sources of misinformation | Focuses on identifying common characteristics of misinformation (including exaggeration, naming, misappropriation, using simplified Chinese and specific terms) and understanding potential sources of misinformation. | "This rumor is in simplified Chinese, with terminology mainly used in Mainland China, not commonly used in Taiwan." "Media reports presenting one-sided facts can easily mislead." "Falsely using authority figures’ or celebrities’ names is a common form of misinformation." "Satirical articles from comedy websites are misleadingly used to deceive people." |
|  | Misinformation early warning and prediction | Recent warnings related to misinformation are provided by identifying and understanding the background mechanisms of the spread of misinformation. | "With the weather cooling down, stroke prevention rumors become a topic of concern." "Rumors about 'how to prevent the virus' and 'vaccine safety' don't just disappear after being debunked, but resurface with every local outbreak, repeatedly appearing online." |
| **Value** in health promotion yes: 1, no: 0 | Health promotion and health literacy | Guides personal healthcare, covering topics such as nutrition, disease prevention, and healthy habits for everyday living. | "A balanced diet, normal routine, and regular exercise can boost immunity." |
|  | Positive attitude toward pandemic prevention | Involves preventing and controlling infectious diseases, including wearing masks, maintaining social distancing, frequent handwashing, and getting vaccinated. | "Let us be considerate of frontline epidemic prevention staff, help spread the word, continue positive epidemic prevention, and jointly fight the virus." |
|  | Media or eHealth literacy | Emphasizes the importance of developing the ability to identify and handle information and providing practical advice, such as fact-checking tools and techniques. | "Understanding 'celebrities are not necessarily experts' in science and media literacy to filter true and false knowledge, be a smart consumer of information." |
|  | Public trust | Highlights the professionalism and responsibility of governments and organizations in maintaining public health and providing accurate information. | "The government also strictly monitors vaccine safety, the public can be reassured to get vaccinated." "77 fact-checking organizations form a global COVID-19 fact-checking front, jointly verifying misinformation and exchanging the latest information since January 24." |
|  | Psychological well-being and social confidence | Emphasizes maintaining public mental stability and societal confidence during public health crises, social events, or other important moments. This includes positive, reassuring messages conveyed by governments, experts, or the media to reduce unease and promote a positive and harmonious social atmosphere. | "The public should view the movement data of confirmed cases without probing into others' privacy or harboring blame." "Thus, the public can safely use products with a license number." |

Table S3. Verbatim coding scheme.

| **Verbatim coding scheme** | **Description** | **Example** |
| --- | --- | --- |
| **Numeric** and statistical information as a basis for correction yes: 1, no: 0 | The correction provides numerical data or statistical information as the basis for correcting and debunking misinformation (excludes numerical data that does not support clarification or description of misinformation). | "The evidence indicates that approximately 80% to 90% of patients can maintain the stability of their condition, and 30% to 40% can even improve their vision." |
| **Authority** from named experts, scholars, or official institutions used as references yes: 1, no: 0 | The article mentions "named experts and scholars" or "professional and official organizations" (excluding self-citations). | "Dr. Mei-Hsiang Ho, an adjunct researcher at the Institute of Biomedical Sciences at Academia Sinica, explained…" |
| **Facts** presented with varying levels of detail; text with no correction: 0, simple correction: 1, detailed correction: 2. | The level of detail in providing factual information. Some articles are not corrections directed at specific objects but may serve as reminders or warnings about misinformation. Some articles involve simple corrections, while others provide detailed content. Characteristics of detailed articles typically include a structured format with background information, fact-checking, points of contention, and conclusions. |  |

Table S4. Prompt for identifying categories of user engagement in comments.

| Given the following social media comments, classify each comment into four categories: 'Knowledge-based Engagement,' 'Critical Engagement,' 'Bias-based Engagement,' and 'Emotional Engagement.' Additionally, for comments categorized as 'Emotional Engagement', specify whether they are positive, negative, or neutral in tone.  Knowledge-based Engagement' if it contains informed, rational, or constructive input.  Critical Engagement' if it contains criticism, skepticism, or mockery that challenges, debunks, or condemns misinformation, rumors, or bias. This category can also include comments that are sarcastic or use humor to challenge misinformation or rumors.  Bias-based Engagement' if it contains misleading information, rumors, or baseless claims without any intention of critical evaluation or debunking.  Emotional Engagement' if it primarily expresses emotions or personal sentiments not necessarily targeted towards misinformation. For comments in this category, further classify them as 'positive,' 'negative,' or 'neutral' based on the sentiment they express.  Example:  Comment: ' We don't use the word; it looks problematic at first glance.  Classification: 'Critical Engagement' (It expresses skepticism about the use of a particular word, suggesting that the word itself could be problematic or inappropriate, thereby challenging its usage in a context that implies criticism.) |
| --- |

Table S5. Examples of output generated by GPT-3.5 for identifying user engagement in comments.

| **Code** | **Output Example (GPT-3.5)** |
| --- | --- |
| Knowledge-based Engagement | "香菜不能治百病Cilantro cannot cure all diseases."  This comment is providing a factual statement that cilantro cannot cure all diseases, which seems to be an informed and rational input. |
| Critical Engagement | "香菜謠言每幾個月就會有新的New cilantro rumors emerge every few months."  This comment appears to be expressing skepticism about the recurring nature of rumors regarding cilantro, indicating that new rumors keep emerging every few months. |
| Bias-based Engagement | "香菜水可以治療腎衰竭 Cilantro water can treat kidney failure."  This comment claims that water infused with cilantro can cure kidney failure. Since there is no scientific basis for this claim. |
| Positive Emotional Engagement | "感謝，香菜好吃Thanks, cilantro is delicious."  This comment expresses gratitude and a positive opinion about the taste of cilantro. |
| Negative Emotional Engagement | "香菜噁心斃了滾Cilantro is disgustingly gross, get lost."  This comment expresses strong dislike and a negative opinion about cilantro. |
| Neutral Emotional Engagement | "我以為會有香菜I thought there would be cilantro."  This comment seems to express a neutral or indifferent personal expectation or observation. |
